# Music-Lessons

**Published:** 1873-11

**Authors:** 


**Music-Lessons.**—Porpora, one of the most illustrious composers of Italy, entertained a great feeling of friendship for a young man, a pupil of his. He asked his youthful acquaintance whether he thought he possessed courage enough to follow constantly the road he, Porpora, traced out for him, however wearisome it might appear. On receiving an affirmative reply, Porpora wrote down, upon a piece of ruled paper, the diatonic and chromatic scales, both ascending and descending, skips of thirds, fourths, fifths, etc., to teach him to master the intervals and sustain the sound, besides shakes, groups, *appogiaturi*, and other vocal exercises of various kinds. This one sheet of paper furnished both master and pupil occupation for a year; the following year also was devoted to it. The pupil began to murmur, but the master reminded him of his promise. The fourth year passed, the fifth year followed, and still there was the same eternal sheet of paper. Even during the sixth year it was not given up, though lessons in articulation, pronunciation, and declamation were added. At the end of the year, the pupil, who thought he was only engaged on the elements of his art, was surprised at hearing his master say: "There, my dear boy, you have nothing more to learn; you are the first singer in Italy." Porpora spoke the truth, for the singer was Caffarelli.

